# Randomised controlled trial of a complex intervention by primary care nurses to increase walking in patients aged 60–74 years: protocol of the PACE-Lift (Pedometer Accelerometer Consultation Evaluation - Lift) trial

**DOI:** 10.1186/1471-2458-13-5

**Published:** 2013-01-04

**Authors:** Tess Harris, Sally Kerry, Christina Victor, Ulf Ekelund, Alison Woodcock, Steve Iliffe, Peter Whincup, Carole Beighton, Michael Ussher, Lee David, Debbie Brewin, Fredrika Adams, Annabelle Rogers, Derek Cook

**Affiliations:** 1 Population Health Research Centre, St George’s University of London, London SW17 ORE, UK; 2 Pragmatic Clinical Trials Unit, Queen Mary’s University of London, London, E12AT, UK; 3 Gerontology and Health Services Research Unit, Brunel University, London, UB8 3PH, UK; 4 MRC Epidemiology Unit, University of Cambridge, Cambridge, CB2 OQQ, UK; 5 Department of Sport Medicine, Norwegian School of Sport Sciences, PO Box 4014, Oslo, 0806, Norway; 6 Psychology Dept, Royal Holloway, University of London, London, TW20 OEX, UK; 7 Department of Population Health Sciences, University College, London, NW3 2PF, UK; 8 Faculty of Health and Social Care, London South Bank University, London, SE1 0AA, UK; 9 10 Minute CBT, Devonshire Business Centre, Letchworth Garden City, Herts, SG61GJ, UK

**Keywords:** Physical activity, Older people, Pedometers, Accelerometers, Walking intervention, Cognitive behavioural, Primary care, Practice nurse

## Abstract

**Background:**

Physical activity is essential for older peoples’ physical and mental health and for maintaining independence. Guidelines recommend at least 150 minutes weekly, of at least moderate intensity physical activity, with activity on most days. Older people’s most common physical activity is walking, light intensity if strolling, moderate if brisker. Less than 20% of United Kingdom 65–74 year olds report achieving the guidelines, despite most being able to. Effective behaviour change techniques include strategies such as goal setting, self-monitoring, building self-efficacy and relapse prevention. Primary care physical activity consultations allow individual tailoring of advice. Pedometers measure step-counts and accelerometers measure physical activity intensity. This protocol describes an innovative intervention to increase walking in older people, incorporating pedometer and accelerometer feedback within a primary care nurse physical activity consultation, using behaviour change techniques.

**Methods/Design:**

Design: Randomised controlled trial with intervention and control (usual care) arms plus process and qualitative evaluations.

Participants: 300 people aged 60–74 years registered with 3 general practices within Oxfordshire and Berkshire West primary care trusts, able to walk outside and with no restrictions to increasing their physical activity.

Intervention: 3 month pedometer and accelerometer based intervention supported by practice nurse physical activity consultations. Four consultations based on behaviour change techniques, physical activity diary, pedometer average daily steps and accelerometer feedback on physical activity intensity. Individual physical activity plans based on increasing walking and other existing physical activity will be produced.

Outcomes: Change in average daily steps (primary outcome) and average time spent in at least moderate intensity physical activity weekly (secondary outcome) at 3 months and 12 months, assessed by accelerometry. Other outcomes include quality of life, mood, exercise self-efficacy, injuries. Qualitative evaluations will explore reasons for trial non-participation, the intervention’s acceptability to patients and nurses and factors enhancing or acting as barriers for older people in increasing their physical activity levels.

**Discussion:**

The PACE-Lift trial will determine the feasibility and efficacy of an intervention for increasing physical activity among older primary care patients. Steps taken to minimise bias and the challenges anticipated will be discussed. Word count 341.

**Trial registration number:**

ISRCTN42122561

## Background

*Why is physical activity important for older people*? The United Kingdom (UK) Chief Medical Officers recently published a report on physical activity (PA) for health, which has drawn upon recent international large scale reviews of the evidence of the impact of physical activity on health and included a specific chapter on older adults [1]. The following benefits for older adults are described: reduced mortality; a reduced risk of over 20 diseases and conditions (including cardiovascular disease, diabetes, obesity, osteoporosis, several cancers, depression, dementia); reduced falls risk; and improved function, quality of life and emotional well-being [1]. These effects are of major importance to both older people and society, with the annual direct cost of physical inactivity to the National Health Service (NHS) across the UK recently estimated at £ 1.06 billion [1].

*What are the physical activity guidelines for older people and how much do older people actually do*? Older adults are advised to be active daily and over a week their activity should add up to at least 150 minutes (2 ½ hours) of moderate intensity activity in bouts of 10 minutes or more, for optimum health benefits. One effective way to achieve this is to do 30 minutes moderate intensity activity on at least 5 days a week [1-3]. Moderate intensity PA makes you warm and increases breathing and heart rate, but should allow talking. Regular walking is the commonest PA of older adults, walking at a moderate pace of 3 miles (5 km) /hour expends sufficient energy to qualify as moderate intensity PA [4]. Faster walking speeds are associated with reduced mortality in older adults [5]. Both adults and older adults are also advised to minimise the amount of time spent being sedentary (sitting) for extended periods [1]. UK public health policy now has an emphasis on helping older adults to increase their physical activity, particularly walking [1,6,7]. These policies are reflected in local initiatives such as Health Walks and GO Active (Get Oxfordshire Active). However, less than 20% of 65–74 yr olds in England report achieving these recommended physical activity levels [8]. The majority of older adults not walking can do so [9]. Since walking is unreliably recalled [10], surveys may overestimate physical activity. Objective accelerometer measurement found only 2.5% of those aged 65 and over [11] achieved recommended physical activity levels.

*How can older adults increase their physical activity*? A critical review and a best practices statement on older adults’ physical activity interventions advised home rather than gym-based programmes and behaviour change techniques (e.g. goal-setting, self-monitoring, building self-efficacy and social support and relapse prevention) rather than health education alone [12,13]. These and other effective behaviour change techniques are emphasized in the NHS Health Trainer Handbook, based on several theories of health behaviour change and intended for NHS behaviour change programmes, with local adaptation [14]. National Institute for Health and Clinical Excellence (NICE) public health guidance on behaviour change also concluded that the evidence did not support any particular model of health behaviour change and that training should focus instead on generic competencies and skills rather than specific models [15]. Starting low, but gradually increasing to moderate intensity is promoted as best practice, with advice to incorporate interventions into the daily routine (e.g. walking) and to monitor intensity for progression [13]. A recent systematic review of interventions to promote walking concluded that interventions tailored to people’s needs, targeted at the most sedentary or at those most motivated to change and delivered at the individual or household level, can encourage people to walk more [16].

*What are the risks from increasing physical activity for older people*? Whilst there are risks for older people associated with regular physical activity, the risks of a sedentary lifestyle far exceed them [13]. Moderate intensity physical activity carries a low risk of injury [17], the commonest adverse events are musculoskeletal injury or falls [18]. Walking appears to be very low risk and has been described as a near perfect exercise [4]. Screening all participants before taking part in physical activity programmes is no longer advocated, as there is a very low degree of risk for light to moderate intensity physical activity [19], but the best practice statement advises that older should have risk management strategies for prevention of activity-related injuries; the most important being to start with low intensity physical activity and increase intensity gradually, the “start-low-and-go-slow” approach [13,20].

*Can activity monitors help to increase physical activity*? Pedometers provide direct feedback on physical activity frequency (step-counts); accelerometers require computer analysis but also provide a time-stamped record of step-counts and physical activity intensity (activity counts) for feedback about specific activities. A systematic review found pedometer users increased steps/day by 2491(1098–3885) and physical activity levels by 27%, but information on older adults was limited [21]. A recent, larger American randomised controlled trial (RCT) (n = 147) in older adults showed an increase of 1320 steps/day at 12 weeks [22]. A Scottish pedometer study of 210 older women found that physical activity was increased at 3 months by a pedometer plus behaviour change intervention (BCI), but the provision of pedometers yielded no additional benefit to the BCI apart from reducing drop-outs, and increased physical activity was not sustained at 6 months [23]. A further Scottish study recruited 41 older adults into a primary care pedometer programme and found that step-counts were significantly increased from baseline to week 12 and maintained at week 24, with associated improvements in quality of life and reduced sedentary time [24,25]. Two other recent trials in older high risk patients (cardiac patients n = 65, and impaired glucose tolerance n = 87) have shown sustained increases in step-counts at 12 months [26,27]. Our study is larger than any other pedometer intervention with older adults and has longer follow-up than other community based studies. The Scottish studies have measured outcomes with accelerometers, but have not fed back accelerometer information to participants. Two small studies showed promising results from feedback, but neither included older adults [28,29]. Accelerometers are acceptable to older adults [11,30] and can monitor physical activity intensity, as advised [13]. Our previous work with older people using these monitors demonstrated that 99% (238/240) of those recruited provided data on at least 5 full days of wear (>10 hours) after 7 days of monitoring [11].

*What is primary care*’*s role*? Primary care is accessible and offers continuity of care for older people, with many chronic diseases being an indication for increasing physical activity. New NHS Health Checks being rolled out in primary care by 2013, include adults up to age 74 and incorporate advice on increasing physical activity, particularly regular walking, often by primary care nurses [31]. Health professional physical activity advice is individually tailored [32] and has greater impact than other advice [33]. Primary care nurses have been shown to be effective at increasing physical activity, particularly walking, in this age group [34]. Physical activity promotion by other routes for this age group is unlikely to be as effective [35]. Recent guidance on prescribing exercise in primary care reinforces the importance of follow-up to chart progress, set goals, solve problems and identify and use social support [36] this will be an important feature of the nurse physical activity consultations in this trial. A large primary care trial is comparing home based with community group exercise [37], but is not examining a primary care pedometer-based intervention. Others have adopted this approach in a small sample with success, but called for a larger trial to confirm findings [25]. Evaluation of the UK Step-O-Meter Programme, delivering pedometers through primary care, showed self-reported physical activity increases, but advised investigation with a RCT design [38].

*Rationale*: A pedometer-based walking intervention, delivered through primary care nurse physical activity consultations, based on established behaviour change techniques and with 12 month follow-up, needs testing in older adults. Accelerometer use, to assess physical activity outcomes and to provide feedback on physical activity intensity as an intervention, in addition to pedometer feedback, also requires testing.

*Study aims*: The main hypothesis to be addressed is whether or not an intervention based on pedometer and accelerometer feedback combined with practice nurse physical activity consultations in primary care is effective in helping people aged 60–74 years to increase their physical activity levels over a three month period and to maintain any increase over a year. The study will also assess whether any effect is modified by age, gender or taking part as a couple and will estimate the effect of the intervention on patient reported outcomes and anthropometric measures.

## Methods/Design

This paper was written according to CONSORT reporting guidelines for randomized trials of non-pharmacologic treatment [39].

### Trial design

**Figure 1:**
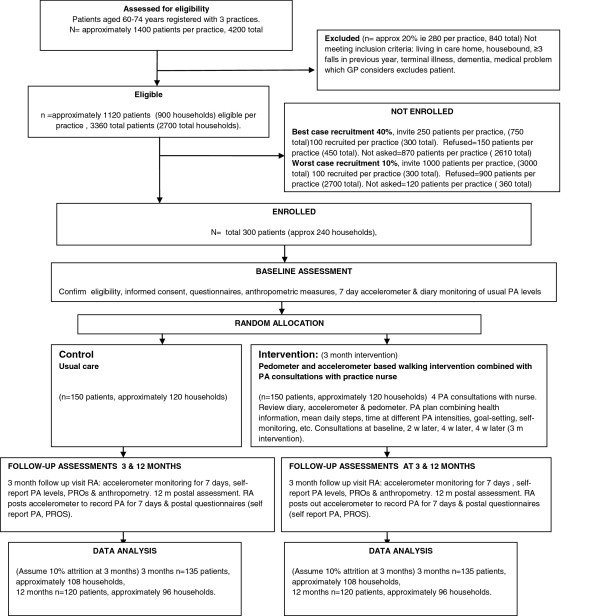
**CONSORT diagram for PACE**-**Lift showing participant flow through each stage of the randomized trial.**

A 2 arm parallel design cluster randomised controlled trial with household as the unit of randomisation comparing a complex intervention to increase walking by primary care nurses with a usual care control group. A 1:1 allocation will be used. There are additional process & qualitative evaluations. The CONSORT diagram [39] summarises the design (Figure 1).

### Practice and participant recruitment

The study will be based in three practices in Oxfordshire or Berkshire West Primary Care Trusts (PCTs) who can commit to participate over the duration of the study. Each practice needs to have the following: a list size >10,000 patients or >1400 patients aged 60–74 years; a practice nurse interested in carrying out the physical activity interventions; and the availability of a room for the research assistant.

Patients aged 60–74 years registered at one of the 3 general practices who are able to walk outside the home and have no restrictions to increasing their physical activity will be eligible to take part. Patients will be excluded if they are living in a residential or nursing home; housebound; ≥3 falls in previous year or ≥1 fall in previous year requiring medical attention; terminal illness; dementia or significant cognitive impairment (unable to follow simple instructions); registered blind; new onset chest pain, myocardial infarction, coronary artery bypass graft or angioplasty within the last 3 months; medical or psychiatric condition which the general practitioner (GP) considers excludes the patient (e.g. acute systemic illness such as pneumonia, acute rheumatoid arthritis, unstable or acute heart failure, significant neurological disease or impairment, unable to move about independently, psychotic illness).

The number of patients aged 60–74 years will be recorded at each practice. Practice staff will search practice electronic primary health care records to identify patients aged 60–74, using Read codes supplied by researchers and local care home knowledge to screen out ineligible patients (as above). A list of potentially eligible patients will be created, these need to be ordered by household (i.e. if there are 2 members of a couple living at the same address who are both potentially eligible this will be a double household; if there is only one person potentially eligible this will be a single household). A random number list will then be used to select the sample of households to be approached after exclusions have been made. Initially a random sample of 200 households containing eligible patients will be selected at each practice, the list will be examined by a practice GP to ensure trial suitability. Patients in these households will then be mailed an invitation letter about the trial from the practice. A follow-up invitation will be mailed out to those who do not respond after approximately 6 weeks. Further random samples of households will be selected from the list at 3 monthly intervals until required numbers have been randomised (100 individuals in total to intervention and control groups in each practice). On the reply slip, those not wishing to take part in the trial will be asked about their willingness to fill in a questionnaire about their health, physical activity levels and reasons for not wanting to participate. Patients who are interested in participating in the trial will be telephoned to arrange a baseline assessment at the practice with the research assistant. The research assistant will post out the Participant Information Sheet, so that they have time to read it before attending the baseline assessment. Two eligible people within a household will be invited together (or apart if they prefer). Eligibility will be confirmed and written informed consent sought at the appointment with the research assistant, before any trial related procedures are undertaken.

### Qualitative evaluation

A qualitative evaluation will run parallel to the trial and will focus upon three distinct groups. i) Trial ‘non-participants’ who agree to be interviewed, to explore factors influencing their decision not to participate. ii) Purposive samples of trial participants, after 12 month follow-up, including samples of those who did not increase PA and those who did. The samples will reflect the range of socio-demographic characteristics of older people. iii) All practice nurses (maximum 6 if two part-time practice nurses per practice) will be invited to participate in semi-structured interviews focussing upon their evaluation of the interventions’ acceptability and use in PA consultations. Interviewing for the older people will continue until no new themes are identified (approx 45 are anticipated for the older people, 15 for the ‘non-participants’ and 15 for each of the trial participant groups)

### Baseline assessment

The following assessments will be carried out by the research assistant at the patient’s general practice.

i) Questionnaire measures: Self-reported physical activity: [modified Zutphen [40]]. Health problems and lifestyle factors: self-reported chronic diseases (e.g. heart disease, lung disease, arthritis, depression etc.); disability [Townsend Disability Score [41]]; medication; smoking; and alcohol. Patient Reported Outcomes (PROs) [Geriatric Depression Scale-15 [42]; anxiety [43]; exercise self-efficacy [44]; perceived health status, including pain, EuroQol 5D (EQ-5D) [45]; and loneliness [46]]. Socio-economic-demographic measures: self-reported ethnic group; marital status; employment; and home ownership. A further self-report questionnaire of 7 day physical activity recall [General Practice PA Questionnaire (GPPAQ) [47] and International Physical Activity Questionnaire (IPAQ) [48]] will be completed after wearing the physical activity monitors for 7 days and handed or posted back with them.

ii) Falls Risk Assessment Tool (FRAT) [49] will be assessed from self-report items in the questionnaire and by directly observing the ability to rise from a chair of knee height without using their arms

iii) Anthropometric measures - height (measured in bare feet to neared 0.5 cm using a stadiometer); weight (measured to mearest 0.1 kg) body fat, bioimpedence (using Tanita body composition monitor, the same monitor used for baseline and outcome assessments); waist circumference (using standard technique and tape measure with clear plastic slider).

iv) Objective physical activity assessment – research assistant explains to patient how to measure usual physical activity levels for 7 days by wearing an accelerometer (Actigraph GT3X+) on a belt over one hip, all day from getting up in the morning until going to bed at night, only removing for bathing). A diary is also provided to record what activities are done and how long for. Accelerometers and diaries to be handed in at the practice or posted back on completion.

### Randomisation procedure

Participants will return the accelerometers and activity diary to the resaerch assistant at the practice. On receipt the research assistant will check the completeness of the accelerometer data. Participants who do not provide at least 5 days of accelerometers data, of at least 600 minutes per day will not be randomised. They will either be excluded or asked to wear the accelerometers for another 7 days. Participants will be allocated to the trial groups by the research assistant using the Nottingham Clinical Trials Unit internet randomisation service to ensure allocation concealment. Randomisation will be at household level to avoid couple contamination (which could occur if a couple were allocated to different arms). Block randomisation will be used within practice with random sized blocks, varying between 4 and 6, and 1:1 allocation ratio, to ensure balance in the groups and an even workload for nurses. The research assistant will inform participants by telephone whether they have been allocated to the intervention or control group.

### Procedure for the control group

After baseline assessment and randomisation, the research assistant will inform them by telephone of their control status, thank them for participating and arrange a 3 month outcome assessment appointment at the practice, including wearing the accelerometer for a further 7 days. They will receive usual care from the practice, with no other scheduled appointments due to the trial. The research assistant and clerical assistant will arrange postal collection of questionnaire and 7-day accelerometer data at 12 months. On study completion, they will be offered individual feedback on their activity levels from their accelerometer recordings by post along with a pedometer to keep, with instructions on how to use it to monitor their physical activity levels.

### Procedure for the intervention group

The research assistant will inform participants of their intervention status and arrange a nurse appointment (either individually or as a couple, according to patient preference). The nurse will then arrange three further appointments with the intervention participants according to the schedule shown in Table 1. The research assistant will arrange a 3 month outcome assessment appointment at the practice, including wearing the accelerometer for a further 7 days in exactly the same way as for the control group. The research assistant and clerical assistant will arrange postal collection of questionnaire and 7-day accelerometer data at 12 months, as for the control group.

**Table 1:** **Details of the PACE**-**Lift physical activity consultations with the practice nurse**

**Week**	**Sessions**	**Guide to session content**	**Proposed Behavioural Change Techniques (Michie [**[54]])*
**1**	**Session1**: **First steps **(45 minutes) *As soon as possible after randomisation*	Review health status, current activity, health benefits of physical activity	1,2,
		Review baseline accelerometer data (step-counts and time spent in at least moderate intensity PA)	19,
		30 mins × 5 days / week message & how to increase PA safely	4,21
		Teach use of pedometer and calculator to calculate average daily steps	
		Cost-benefit analysis for increasing physical activity	2,
		Ideas of how, when and where to increase step-counts	20,38
		Use of rewards for effort and for achieving goals	12,13
		SMART physical activity goals, weekly walking planner & step-count diary	7,9,16,
		Check confidence levels, ensure goals are realistic	9,
		Summarise & check patient understanding, plan date for wearing accelerometer next and time next meeting	26,
**3**	**Session 2**: **Continuing the changes **(30 minutes) *Approximately 2 weeks after session 1*	Review step-count and accelerometer data and step-count diary	10,19,
		Encourage progress in increasing walking and achieving goals	12,13,
		Troubleshoot any problems with equipment or diary	8,
		Barriers and facilitators to increasing physical activity, overcoming barriers	8
		Review target and set new SMART walking goals using walking planner	7,9,16
		Check confidence levels, build confidence to make change	5,18,29,36,
		Encourage recording in step-count diary	16
		Review and set your own goals	5,8,10,
		Summarise & check patient understanding, plan date for wearing accelerometer next and time next meeting	26,
**7**	**Session3**: **Keeping up the changes **(30 minutes) *Approximately 4 weeks after session 2*	Review step-count and accelerometer data and step-count diary	10,19,
		Encourage progress in increasing walking and achieving goals	12,13,
		Troubleshoot any problems with equipment or diary	8,
		Preparing for setbacks: discussion of coping strategies, building social support	29,35,
		Introduce pacing; general pacing tips and plans	9,35
		Building habits – generate ‘if-then’ plans to prevent setbacks or build habits	7, 23,
		Review target and set new SMART walking goals using walking planner	7,9,16,
		Encourage recording in step-count diary	16,
		Summarise & check patient understanding, plan date for wearing accelerometer next & time next meeting	26,
**11**	**Session4**: **Building lasting habits **(30 minutes) *Approximately 4 weeks after session 3*	Review step-count and accelerometer data and step-count diary	10,19,
		Review overall progress over the sessions	11,
		Encourage progress in increasing walking and achieving goals	12,13,
		Building habits: discuss methods of maintaining lasting change	35,
		Becoming your own activity coach: review benefits of PA	1,2,
		Set a new overall PA target and step-count goal for next 3 months	7,
		Review stages for making change (SMART goals, set rewards, build confidence to change, review goals, overcome barriers, pacing & dealing with setbacks)	7,8,9,12,35

* 1. Provide general information on behaviour-health link; 2. Provide information on consequences to individual; 4. Provide normative information about others’ behaviour; 5. Goal setting (behaviour); 7. Action planning; 8. Barrier identification; 9. Set graded tasks; 10. Prompt review of behavioural goals; 11. Prompt review of outcome goals; 12. Prompt rewards contingent on effort; 13. Prompt rewards contingent on successful behaviour; 16. Prompt self-monitoring of behaviour; 18. Prompting focus on past success; 19. Provide feedback on performance; 20. Provide information on when and where to perform the behaviour; 21. Provide instructions on how to perform the behaviour; 23. Teach to use prompts/ cues; 26. Prompt practice; 29. Plan social support / social change; 35. Relapse prevention / coping planning; 36.Stress management / emotional control training; 38. Time management.

### Components of the complex intervention

i) *Pedometers*. The accuracy of the Yamax Digi-Walker SW-200 (Tokyo, Japan) is high and it is referred to as the criterion pedometer [50-52]. It provides direct step-count feedback to participants, but doesn't measure physical activity intensity. Step-counts need daily manual recording and re-setting.

ii) *Accelerometers*. The Actigraph (GT3X + Manufacturing Technology Inc.,Fl.USA) records the raw acceleration in three axes continuously for up to 40 days depending on the sampling frequency. The output from the accelerometer includes total activity counts, vector magnitude, number of steps accumulated, time spent sedentary and physical activity levels using standard cut-offs [11,30,53]. Accelerometer data require computer processing and analysis, and do not provide any direct feedback to participants.

iii) *PA consultations with a practice nurse*. A guide to the timing of sessions and session content is given in Table 1.

### Providing feedback on physical activity and use of monitors, step-count diary and walking planner

Accelerometer information on each individual will be downloaded on the practice computer by the nurse using the Actilife software (Manufacturing Technology Inc.,Fl.USA ) and the participant shown how much time was spent in sedentary, light, moderate (and possibly vigorous) intensity activities. This will be related to specific activities recorded in their physical activity diary. Average daily step-counts will also be reviewed in this way. For subsequent sessions participants' pedometer step-counts will be used for feedback, but for the first session accelerometer step-counts will be used. (Both groups must have the same accelerometer baseline assessment, as giving the intervention group pedometers would introduce bias). This baseline PA data will be reviewed alongside health & anthropometric data, so that an individual physical activity plan can be produced, tailored to the participant's baseline step-count and time spent in different physical activity intensities, abilities, health, goals and personal circumstances and based on increasing walking and walking speed and other existing physical activities. Participants will be encouraged to set goals relating to both step-counts and time spent at different physical activity intensities, they will be encouraged to start-low-and-go-slow, to minimize risks. The nurse will show them how to use a pedometer including recording their daily step-counts in a diary and using a calculator to calculate average daily step-count. They will also be given a walking planner to help them specifially plan when and where and with whom they plan to walk. They will be asked to wear an accelerometer plus a pedometer and keep a diary record of daily steps for 7 days prior to each consultation, they can wear the pedometer more often if they wish. They will have 3 further consultations over the 3 month intervention, initially fortnightly, then monthly. Their average daily steps and time spent at different physical activity intensities in the 7 days prior to these meetings will be discussed. If they have achieved their goals, new goals can be set, if not, then problems and ways of overcoming them can be discussed.

### Using behaviour change techniques in the physical activity consultation

The nurses will receive training in established, effective behaviour change techniques as emphasised in the NHS Health Trainer Handbook [14] for use in their physical activity consultations with patients. A handbook for patients (Improving Health: Changing Behaviour, PACE-Lift patient handbook, adapted from the NHS Health Trainer Handbook [14], but focusing only on physical activity behaviour change, has been produced for the trial. This will be used for nurse training and by nurses during their sessions with patients, it will also be provided to individual patients to keep at their first appointment, as part of the intervention. The techniques used have been classified according to Michie’s refined taxonomy of behaviour change techniques for physical activity interventions (CALO-RE taxonomy) [54] (see Table 1). Using pedometers and accelerometers and step-count diaries to set goals and monitor progress, as outlined above, should be a useful adjunct to the behaviour change techniques.

### Practice nurse training

Practice nurse training in behaviour change techniques and use of the PACE-Lift patient handbook will be planned and conducted by experienced trainers in behaviour change techniques with primary care and practice nurse training experience (LD and DB) [55]. They will also provide supervision and monitoring to the nurses over the course of the trial, including listening to audio-recordings of a sample of each nurse’s consultations and providing individual feedback. Training will also be provided to the nurses on the use of pedometers and accelerometers and safety aspects of the trial by the Chief Investigator. The fidelity and quality of the implementation of the intervention will be monitored over time and between different nurses by the following methods: i) analysing the content of a sample of audiorecorded sessions for each nurse by the trainers according to an agreed proforma; ii) discussion about consultations during group supervision with all the nurses; iii) completion of a checklist of areas covered in each consultation by the nurse; and iv) completion of a nurse patient alliance questionnaire at the end of each patient’s intervention by both the nurse and the patient.

### Assessment of outcomes after 3 and 12 months in the intervention and control groups

i) *3 month assessment at the patient*’*s general practice*: All components (including acceleormeter assessment) as for baseline assessment, but questionnaire has additional questions about adverse events, including injuries and health problems over the past 3 months.

ii) *12 month postal assessment*: As for baseline assessment (including accelerometer assessment) but there is no anthropometric assessment, and the questionnaire has additional questions about adverse events, injuries and health problems and pedometer use over the past 9 months.

### Outcome measures

The primary outcome is change in average daily step-count between baseline and 3 months. The secondary outcome is change in average time spent in at least moderate intensity physical activity weekly between baseline and 3 months. Both will be assessed objectively by accelerometry as will time spent sedentary. We are also assessing these outcomes at 12 months to determine whether any changes are maintained.

Ancillary outcomes are changes in the questionnaire measures (e.g. depression scores, anxiety, pain, EQ-5D, exercise self-efficacy) assessed at baseline, 3 and 12 months and anthropometric measures assessed at baseline and 3 months. Adverse events (falls, fractures, injuries) will also be assessed. Practice costs will be collected and used with change in health status (EQ-5D) [45] to plan the health economic assessment for a future, larger trial. A qualitative evaluation will examine the acceptability of the intervention and perceived barriers and benefits to physical activity.

### Sample size

A previous pedometer intervention increased steps by around 1000 steps/day at 3 months [22]. 120 participants in each trial arm would allow a difference of 850 steps/day to be detected between the groups with 90% power and 1% significance, using the standard deviation of the difference of 1705 steps/day from pilot work [56]. However, we plan to randomise households. Assuming an intracluster correlation of 0.5 and that 30% of households recruited are couples, the design effect is 1.15 and we need to analyse 138 people (105 households) at 3 months to achieve the same power. Allowing for approximately 10% attrition at 3 months we aim to randomise 150 people (120 households) into each arm, giving a total of 300 people. 100 people from each of 3 practices will be recruited over 12 months, a target of 9 patients per month per practice. We are intending to recruit from practices with list sizes ≥ 10,000. Each practice will have approximately 1400 patients aged 60–74 of whom we anticipate 80% will be eligible. We will select patients at random to take part until required numbers have been randomised.

### Anticipated recruitment

Our observational study of PA in older primary care patients had 43% recruitment [57], but primary care PA intervention studies with older people have lower recruitment: 17% [58] 35% [59] 14% [60]. We anticipate 30% recruitment, but even if 10%, we could still randomise the required numbers (Figure 1).

### Statistical analysis

Analysis and reporting will be in line with CONSORT guidelines, with primary analyses being on an intention-to-treat basis, that is, all participants will be included who have outcome data, regardless of their adherence to the intervention. Sensitivity analyses including all randomised patients will be carried out using multiple imputation.

Descriptive statistics will be used to assess any marked baseline imbalance in prognostic variables; age, gender, ethnicity, season, social deprivation (occupational pension (yes/no) and difficulty paying bills), Geriatric Depression Scale, perceived health status, Townsend Disability Score, weight, BMI, and percentage body fat. The same variables will be compared between those who complete follow up and those who drop out completely, and those who fail to provide a complete set of five days data for the primary outcome. Significance tests, either t-test or chi-squared tests, will be used to compare those with complete data and those who have missing outcomes.

### Primary analysis

The primary outcome measure is change in step count from baseline to 3 month follow up. Secondary measures which we will also examine are counts per minute, counts per minute of registered time and number of minutes spent in moderate or vigorous physical activity. These measures are likely to be highly correlated with step count and will be analysed using identical approaches to that for step count.

The primary analysis will use all patients with outcome data (i.e. complete case analysis, adjusted for covariates likely to predict outcome and missingness). The main outcome will be the average number of steps per day measured over seven days at three months. By including baseline number of steps per day as a covariate, this will effectively be measuring change in number of steps over the three months. Baseline value of the outcome, practice, season, whether or not taking part as a couple, body mass index, disability and perceived health status will be included as covariates in all analyses. Additionally the patient level covariates will include any variable in the descriptive analysis found to differ markedly between the two intervention arms and any of these variables found to predict missingness. Daily step counts will be analysed using a multi-level random effect regression model allowing for clustering at household level, to compare participants receiving the pedometer and accelerometer based intervention with those receiving usual care. At the day level, day of the week will be added as a covariate to adjust for day of the week, which is particularly important if some days are missing.

### Secondary analyses

Adverse events: Numbers in each group who have suffered a fracture, falls and injuries and drop-outs will be compared between the groups using logistic regression in Stata adjusted for clustering

Subgroup analyses: Interaction terms will be added to the regression model to test whether the intervention effect varies between men and women, between different age groups (60–64, 65–69, 70–74) and between those taking part as a couple and those taking part individually.

Dose effect relationship: The number of follow-up intervention sessions (1,2 or 3) attended will be added to the model to investigate the effect of compliance on outcome.

### Stopping rules

It would be impossible to carry out interim analyses on sufficient patients to decide to stop, so there are no formal statistical stopping rules. If a patient becomes ineligible, the nurse may discontinue the intervention, but all patients will be asked to complete follow-up assessments. Patients can withdraw at any time.

### Procedure for accounting for missing, unused and unexpected data

Only days with ≥600 minutes of registered time on accelerometer on a given day will be used. Participants will only be randomized if they provide at least 5 such days of accelerometer data at baseline. Therefore missing baseline data will not be a major problem at baseline but we will use a multilevel linear regression model, taking account of clustering within household and repeat days within individuals to estimate the baseline level for each subject, adjusted for day of the week and days since start of measurement. The potential for missing data at follow-up will be also be reduced. The three main covariates, practice, season and whether or not taking part as a couple will be known for all participants, and it is anticipated that most patients will have complete data for other measures. If any covariates have missing values, these will be replaced by the mean value [61]. Multi-level modeling will be used to allow for day of week of measurements. This will allow change within subjects to be estimated, even if less than 7 days of data are provided.

The main analysis assumes that, conditional on the model covariates, outcome data are missing at random. This is likely to be true for missing data due to accelerometer failure, and is plausible for missing days and participants who do not return accelerometers. However an alternative plausible assumption is that participants who fail to provide outcome data are less active. Multiple imputation analysis adjusting the imputed values downwards by a range of values will assess the sensitivity of the analysis to the missing at random assumption [61].

### Participant withdrawal

Participants will be free to withdraw from the trial at any time and without giving a reason. Practice nurses can advise discontinuation of the physical activity intervention if the intervention poses a hazard to the participant. In both cases, information that has already been collected on participants may still be used and they will be asked if they would be prepared to provide any further data on outcomes at 3 months and 12 months (e.g. questionnaire, anthropometric measurements and/or physical activity monitoring). Withdrawal from the study will not affect the standard of care received from the practice. If participants withdraw before they have been randomised they will be replaced, those withdrawing or being withdrawn after randomisation will not be replaced.

### Adverse event monitoring

A standard operating procedure for the management of adverse events will be in place, so that participants or their relatives, practice staff or researchers can inform the chief investigator of any event they consider possibly related to physical activity promotion. All adverse events reported will be assessed for seriousness, expectedness and causality

### Notification and reporting of adverse events: Retrospective data collection on adverse events

i) *Questionnaires*: Intervention and control groups will be sent questionnaires at 3 months and 12 months that will ask specifically about falls, injuries and exacerbation of any pre-existing conditions in the previous 3 month and 9 month periods respectively.

ii) *Computerised primary care records*: In order to be sure that full data on adverse events is collected, informed consent will be sought to collect data from participants records at the end of the study. All consultation data for the 12 month period of the study for each individual giving consent will be downloaded from practice computerised records, including all new problems recorded during this period. Additionally data on all hospital admissions, out of hours attendances and out patient appointments recorded will be downloaded. This will be anonymised before removal from the practice and a researcher who is blind to the intervention or control status of the participants will analyse this data with a standardised proforma recording possible adverse events.

### Ethical and organizational review

The trial has been reviewed and given a favourable opinion by the Oxfordshire Research Ethics Committee C (11/H0606/2). Research and development approval has been granted by Oxfordshire and Berkshire West PCTs.

## Discussion

The PACE-Lift trial is a primary care based physical activity intervention for 60–74 year olds which seeks to discover if the combination of feedback from pedometers and accelerometers plus consultations with a nurse based on behaviour change techniques is feasible and if it can increase physical activity levels. It is a pragmatic trial being conducted across several general practices with patients’ own practice nurses, rather than trained researchers or therapists delivering the intervention. The findings will therefore be of direct relevance to UK primary care.

We have taken the following measures in the trial to minimise or avoid bias:

1. Randomisation: The Nottingham Clinical Trials Unit internet randomisation service will be used to ensure allocation concealment. Randomisation will be at household level to avoid couple contamination (see below).

2. Contamination: Contamination could occur between partners in the same household, we will minimise this by ensuring that if both are recruited they are allocated to the same group. Contamination could also occur in the controls if they seek to increase their physical activity. We will try and discourage the control group from buying a pedometer by ensuring that they know that they will receive one, along with instructions on its use and feedback on their individual activity levels at the end of the trial. The 3 month and 12 month assessments will capture information on physical activity in the control group, including a question in the 12 month assessment about whether they have used a pedometer at all in the previous 12 months.

3. Blinding and assessment of outcomes: Participants cannot be blinded to their intervention or control status. The research assistants assessing outcomes will not be blinded to the participants’ intervention status for pragmatic reasons; the study is funded to support only two research assistants to carry out recruitment and follow-up simultaneously at their allocated practices. Appointments for the 3 month and 12 month outcome assessments will be booked in advance according to a protocol, taking into account holidays. However, primary and secondary outcome measures are objectively measured by accelerometry and do not rely on assessor interpretation. Physical measurements will also be assessed objectively (e.g. body weight and body fat measurements). Patient reported outcomes will be assessed by validated self-report instruments, minimising researcher bias. The statistician analysing the data will be blind to the treatment allocation of the participants.

The particular challenges that we anticipate in this study are as follows:

1. Low levels of recruitment and possible selection bias, with those who are more physically active being more likely to want to take part. We are addressing this by recruiting from practices with enough older people in the target age range for us to achieve our sample size even if recruitment were as low as 10% of those eligible. In order to estimate response bias we aim to assess self-reported physical activity and health on those who are not recruited to the trial but who are willing to fill out a short questionnaire.

2. Variation in the physical activity intervention delivered across practices and over time. We have several quality assurance mechanisms in place (including recording consultations, group supervision, nurse checklists and patient nurse alliance scales) to help us to avoid and monitor this.

3. Technical difficulties with the equipment, particularly the accelerometers and associated computer software. We plan to try and overcome this by investing adequate time in training the practice nurses in the use of the accelerometers and software and supporting the nurses during the trial, particularly at the beginning when they are getting used to the equipment.

4. Loss to follow-up, particularly of the control group. We hope to reduce this by personal contact with the same research assistant and offering the controls individual feedback on their activity levels after they complete the trial from their baseline, 3 m and 12 m assessments, along with a pedometer and individualized instructions on how they could increase their physical activity levels.

If the trial is well conducted with fidelity of implementation and is feasible, but the intervention is ineffective, then this would be an extremely important negative finding to disseminate to the NHS both locally at practice and PCT level and centrally via National Institute of Health Research mechanisms. It would also suggest that less intensive interventions (e.g. pedometers without support) would be unlikely to be beneficial in this age group.

If the intervention is effective (the intervention group significantly increase their physical activity levels compared with controls and differences are maintained at 12 months) this would be an important positive finding, suggesting an effective intervention to increase physical activity levels that could be delivered through primary care. The qualitative interviews with both patients and nurses will be crucial in helping us to understand what are likely to be the most effective aspects of the intervention. Comparison of the effect size of this trial with another ongoing trial (Harris et al. PACE-UP trial, Health Technology Assessment ISRCTN98538934) which is also being conducted through primary care, includes the same age group and is using the same baseline and outcome measures, but with a simpler intervention (pedometers alone or pedometers plus nurse support, but no accelerometer feedback) will also be useful in comparing which aspects of the intervention are necessary for increasing physical activity levels in this age group.

If the intervention is effective at 3 months, but not at 12 months, then further work on how to maintain increases in physical activity levels in this age group would be indicated. 

## Abbreviations

UK: United Kingdom; PA: Physical activity; NHS: National Health Service; NICE: National Institute for Health and Clinical Excellence; RCT: Randomised controlled trial; BCI: Behaviour change intervention; PCTs: Primary Care Trusts; GP: General practitioner.

## Competing interests

The authors declare that they have no competing interests.

## Authors’ contributions

TH, DC and CV conceived the idea for the study. TH, DC, CV, SK, AW, SI, UE, PW, CB and FA participated in the design of the study and developed the research protocol for funding. SK and DC were responsible for the statistical analysis plan and the sample size calculations. CV, AW, TH and AR designed the qualitative aspects of the study. TH, AW, LD, DB and MU designed the behaviour change intervention, adapted the NHS health trainer handbook for the purposes of the trial and designed the nurse training. FA and AR were also involved in compiling patient information and data collection packs. All the authors have read and approved the final manuscript.

## Pre-publication history

The pre-publication history for this paper can be accessed here:

http://www.biomedcentral.com/1471-2458/13/5/prepub
